# Passive Heat Stimuli as a Systemic Training in Elite Endurance Athletes: A New Strategy to Promote Greater Metabolic Flexibility

**DOI:** 10.3390/jfmk10020220

**Published:** 2025-06-07

**Authors:** Sergi Cinca-Morros, Martin Burtscher, Fernando Benito-Lopez, Jesús Álvarez-Herms

**Affiliations:** 1 Microfluidics Cluster UPV/EHU, Analytical Microsystems & Materials for Lab-on-a-Chip (AMMa-LOAC) Group, Analytical Chemistry Department, University of the Basque Country UPV/EHU, 48013 Bilbao, Spain; scinca2@gmail.com (S.C.-M.); fernando.benito@ehu.eus (F.B.-L.); 2 Microfluidics Cluster UPV/EHU, BIOMICs Microfluidics Group, Lascaray Research Center, University of the Basque Country UPV/EHU, 01006 Vitoria-Gasteiz, Spain; 3 Department of Sport Science, University of Innsbruck, A-6020 Innsbruck, Austria; martin.burtscher@uibk.ac.at; 4 Physiology and Molecular Laboratory (Phymolab), 40170 Collado Hermoso, Spain

**Keywords:** passive hyperthermia, thermoregulation, metabolic flexibility, aerobic performance, endurance athletes, physiological responses

## Abstract

**Objectives:** The ability to efficiently regulate body temperature is crucial during endurance activities such as trail running, especially during competitive events in hot conditions. Over the past decade, passive hyperthermia exposure has grown significantly in popularity as a means of improving acclimatization and performance in hot environments. The present study aims to compare the physiological changes that occur in a group of professional athletes due to passive sauna exposure (80–90 °C) and their own response to maximal aerobic performance. **Methods:** Twelve professional trail runners (eight men and four women) were tested in three conditions: (i) baseline; (ii) before; and (iii) after (a) passive dry sauna exposure and (b) a maximal endurance test. In both cases, physiological parameters such as heart rate, tympanic temperature, arterial and muscle oxygen saturation, and blood concentrations of glucose, total cholesterol, high-density lipoprotein (HDL) and hemoglobin were measured. **Results:** Sauna exposure produced similar trends in cardiovascular and metabolic responses to those occurring during exercise, but at a much lower physiological level. Glucose and HDL levels were both significantly elevated (or tended to be so) after sauna and exercise (*p* < 0.03 and *p* < 0.01, respectively). Athletes who mobilized the sum of substrates (glucose and HDL) performed the exercise test faster (r = −0.76; *p* < 0.004). The response of arterial oxygen saturation (decreased) was similar during sauna and exercise, but opposite at the muscular level (increased during sauna and decreased during exercise). Additionally, inter-individual variability in responses was noted for most of the other parameters, suggesting the existence of ‘responders’ and ‘non-responders’ to thermal stimuli. **Conclusions**: The physiological responses of trained endurance athletes are moderately impacted by passive sauna use. However, individual changes could be correlated with endurance performance and optimizing individualization. Heat stimuli promote different physiological responses in terms of cardiac function, oxygen kinetics and substrate mobilization, albeit to a lesser extent than exercise. Greater substrate mobilization during maximal endurance exercise was found to be correlated with better performance. Further studies are needed to explore the concepts of metabolic flexibility, as described here, and how heat exposure may improve systemic health and performance.

## 1. Introduction

The thermoregulatory process is vital for humans but its biological cost is very high and involves various organic systems [1]. During the last few decades, climate change has caused increasingly extreme thermal environmental conditions, such as higher temperatures, humidity, airflow, and solar radiation [2].

Extreme heat conditions that occur during the summer impair endurance performances due to increased physiological demands [2]. It has been reported that endurance exercise, such as during marathons or ultra-endurance events, comprising 24 h or longer, performed in heat conditions acutely alters body composition [3], reduces aerobic capacity [4], increases cardiovascular strain [5], raises perceived exertion, decreases motivation [6] and promotes the premature depletion of endogenous glycogen stores due to a greater reliance on the anaerobic metabolism [7] and hormones [8,9]. At present, over a quarter of total endurance events are carried out in moderate, high and extreme heat conditions, with this number even reaching one half if marathons are excluded [10]. In this regard, during the last decade, the number of publications dealing with heat stimulus as a training and acclimatization method (passive and active) has grown significantly [11]. Subsequently, athletes and coaches are becoming more and more conscious of the specific importance of including heat training during seasonal preparations as a strategy to improve performance in heat [12].

The number of participants in trail running competitions has grown exponentially over the last few decades. The majority of trail and ultra-endurance competitions take place during the summer, when extreme heat directly impairs physiological performance. In fact, high temperatures increase the risk of dehydration, gastrointestinal distress and electrolyte imbalance [13]. Original research by Pugh et al. [14], published in 1967, was the first to hypothesize that higher tolerance to hyperthermia was related to better success in ultra-marathoners. This hypothesis was confirmed by other studies which also demonstrated that greater dehydration tolerance was beneficial for better endurance performances during heat [15,16,17]. Ultra-endurance trail running promotes important systemic impacts at different systemic levels, including metabolism, oxygenation uptake, neuromuscular fatigue and cognitive functions [18]. Therefore, the better tolerance of hot conditions directly benefits performance and health during trail and ultra-endurance running competitions.

Previous studies have proposed different protocols for monitoring physiological changes that occur during heat exposure in athletes [19,20]. However, there is no clear scientific consensus on the individual thermal dose or type of response (passive or active) to prescribe heat stimuli. It is reasonable to expect that there is an individual heat tolerance threshold related to genetic predisposition that enhances adaptive epigenetic plasticity when athletes are exposed to heat over a long period of time [21]. Here, we propose a new method of monitoring individual changes in different physiological responses by comparing the responses to passive heat stimuli and maximal endurance exercise. Unlike previous studies, which measured peak temperatures or isolated metabolic and/or cardiovascular parameters, we hypothesize that a systemic phenotype response exists in response to systemic stimuli such as heat and maximal exercise. To this end, we tested metabolisms through glucose, lipids and lactate; oxygenation capacity through capillary hemoglobin, arterial and muscular saturation; and temperature through skin and tympanic measurements. Overall, the analyses of different systems contributed to the systemic response of the phenotype in elite athletes.

Under this premise, the main goal of the present study was to monitor physiological changes that occurred from acute heat stimulus and maximal physical effort at different systemic levels, namely (i) metabolism, (ii) oxygenation, and (iii) cardiovascular, in non-heat-acclimatized endurance athletes. A systemic threshold of tolerance may be described by a comparison between individuals with themselves after heat and maximal effort. We hypothesized that there are individual physiological profiles of responses to heat exposure that are related to individual exercise responses.

## 2. Materials and Methods

### 2.1. Subjects

Twelve endurance professional trail runners were recruited to participate in the present study (eight men and four women; see Table 1). All participants were considered international athletes with several years of experience in competition. Of the total participants, eight were recruited from a professional trail team (five men and three women) and the other four from other teams (three men and one woman). The present study was approved by the Ethics Committee for Research Involving Human Subjects (CEISH-UPV/EHU, BOPV No. 32, 17 February 2014), with an approval date of 22 January 2024, as well as by the Ethics Committee for Research Involving Biological Agents and/or Genetically Modified Organisms (CEIAB-UPV/EHU, BOPV No. 32, 17 February 2014), with an approval date of 18 September 2023. In the approval document, the main goals of the present investigation were described.

Inclusion criteria were as follows: (a) being an athlete within the top 50 in the world rankings; (b) maintaining systematic training during the 12 months prior to the study; and (c) athletes without any previous heat training experience. There were no exclusion criteria for age (although all were over 18 years of age) or sex. One reason for exclusion from the study was failure to finish the performance or sauna test due to heat intolerance or due to exercise injury or dropout.

Initially, thirteen subjects were recruited; however, one athlete suffered physical problems during the performance test and was excluded from the study statistics. The physiological measurements were obtained during the summer and the pre-competitive phase. Participants trained consistently for at least 12 months prior to studying. All participants were recruited with the consideration that they had not undergone any previous training under hot conditions or using a sauna during their athletic career. Participants provided written informed consent in accordance with the study’s approval by the University of the Basque Country (see Institutional Review Board Statement at final of the manuscript).

### 2.2. Experimental Design

Physiological measurements were taken at baseline (pre-sauna) and immediately after sauna exposure and maximal exercise. Baseline values were obtained in the morning, before breakfast, between 8 and 10 am, for the following factors: heart rate, tympanic temperature, systemic blood pressure, arterial oxygen saturation (SaO_2_), muscular oxygen saturation (SmO_2_), glucose, hemoglobin, total cholesterol, high-density lipoproteins, low-density lipoproteins, triglycerides, and lactate (capillary blood samples from fingers). Anthropometric measures (weight and height) were also taken. Athletes were instructed to eat their most recent meal at least three hours before testing and to avoid stimulant beverages. They were also asked to maintain good hydration and ensure they were well rested.

After obtaining baseline measurements, athletes were individually exposed to passive sauna conditions (80–90 °C) [22] for 20 min or until they reached their tolerance limit. During sauna exposure, athletes were monitored every 2 min for muscle and arterial oxygenation, heart rate, and tympanic temperature, with peak values recorded at the end of the session. Immediately upon leaving the sauna, identical physiological measures were taken again for comparison with baseline values.

A pulse oximeter (Omron model CMS 50D Plus, Qinhuangdao, China) was used to analyze oxygen saturation [23]. Muscle oxygenation was monitored using a portable NIRS (near-infrared spectroscopy) device positioned on the quadriceps (rectus femoris) with a Humon hex device (Boston, MA, USA) [24]. Capillary blood samples were collected from finger tips with hyperemia and analyzed for glucose levels using the Point of Care LUX Meter (Biochemical systems international, Arezzo, Italy) via the amperometry method, for lipids and hemoglobin with the same device via the reflectometric method [25], and for lactate using the Lactate Scout 4 (SensLab GmbH, Leipzig, Germany) [26].

Physiological measures were obtained during a training camp in Chamonix. Physical performance was measured in the mountains immediately after finishing a climb test (a track segment of Strava named “single montée posettes”: https://www.strava.com/segments/15132973?filter=overall, accessed on 19 August 2023), while the passive sauna response was measured in the facilities of the hotel in Chamonix. Forty-eight hours after sauna exposure, all participants performed a maximal (fixed distance) trail running test, consisting of a 1.96 km course with a +419 m elevation gain and an average gradient of 21.3%, a segment published on the Strava platform. Physiological parameters measured during sauna exposure were taken immediately after the trail running test, i.e., heart rate (measured with a chest strap, Garmin CX750 model, Mission, KS, USA), core temperature (measured with a CORE sensor, Zürich, Switzerland), muscle and capillary oxygenation, as well as metabolic and mechanical parameters (as described above). Weather conditions during the running tests ranged from 12 to 22 °C with 40 to 60% relative humidity.

### 2.3. Statistical Analysis

The statistical analyses were conducted using Python (version 3.8) and Jupyter Notebooks (version 6.4.12) for interactive exploration, enabling the dynamic analysis of the dataset. Various statistical methods were employed to compare baseline vs. post-sauna and exercise interventions, including correlation analyses, linear regressions, proportions, and descriptive statistics. Correlation analyses were performed to evaluate a potential association between weight loss (due to sweating in the sauna) and metabolic markers. Paired *t*-tests were employed to compare changes in physiological markers (normally distributed data) between baseline and post-sauna conditions, as well as between baseline and post-exercise conditions. Statistical significance was indicated by a *p*-value ≤ 0.05. The relationship between metabolic substrate mobilization and exercise performance was performed through two combined parameters (glucose and HDL), representing both glycolytic and lipid oxidation pathways. Since glucose and HDL have different units of measure and numerical ranges, direct summation would not be appropriate. To ensure comparability, we applied min–max normalization to each variable across all subjects (both male and female). This transformation scales each parameter between 0 and 1, maintaining relative differences while eliminating unit discrepancies. Two different combined parameters were generated:

1. Combined parameter (Δ Glucose + Δ HDL): Sum of normalized glucose Δ and normalized HDL Δ, where Δ represents the change from baseline to post-exercise.
2. Combined parameter (post-exercise glucose + post-exercise HDL): Sum of normalized post-exercise glucose and normalized post-exercise HDL to assess absolute metabolic values post-exercise rather than relative changes.

## 3. Results

### 3.1. Physiological Responses to Sauna and Exercise

Physiological average data for athletes at baseline, sauna, post-sauna, and post-exercise are presented in Table 1. Statistical analyses of pre- vs. post-sauna changes revealed significant increases in glucose levels (*p* < 0.001) and HDL levels (*p* < 0.05), decreases in SaO_2_ and elevation of SmO_2_ levels (*p* < 0.05), reductions in TG levels (*p* < 0.003), increases in tympanic temperature (*p* < 0.001), and elevations in heart rate (*p* < 0.001) and weight loss (*p* < 0.05).

Maximal exercise provoked increases in glucose levels (*p* < 0.001), elevations in HDL cholesterol (*p* < 0.001), and reductions in hemoglobin levels (*p*< 0.009), LDL cholesterol (*p* < 0.001), SaO_2_, SmO_2_, and maximal temperature reached.

### 3.2. Individual Physiological Responses to Heat Exposure and Running

**Figure 1:**
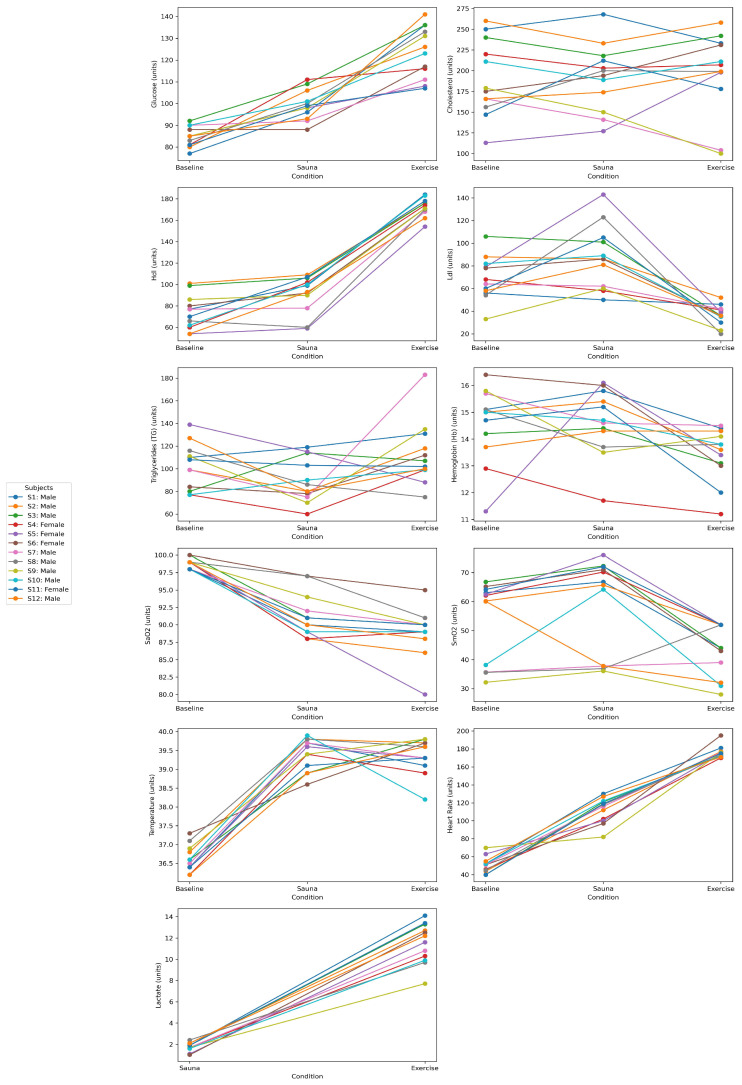
Individual physiological responses to sauna exposure and maximal exercise. Graphs show changes in athletes from baseline (pre-sauna measures) and after/during sauna exposure (sauna conditions) and distance running.

Individual physiological responses to sauna exposure and maximal exercise are illustrated in Figure 1.

Findings showed similarities and differences in physiological responses to heat exposure (sauna) and maximal exercise. Core temperature seems to be more individually regulated than physiological responses to exercise (Figure 1).

### 3.3. Individual Physiological Values

Values of baseline, post-sauna, and post-exercise physiological values can be seen in Table 2. 

#### 3.3.1. Core Temperature

All subjects (Subjects 1 to 12) showed an increase in core temperature under both sauna and maximal exercise conditions (see Table 2). In seven subjects, the temperature increases were greater during the sauna compared to exercise (Subjects 1, 2, 4, 5, 7, 8, and 10), while in five subjects, the increases were lower (Subjects 3, 6, 9, 11, and 12).

#### 3.3.2. Arterial Oxygen Saturation Levels (SaO_2_)

All subjects (Subjects 1 to 12) showed a decrease in arterial oxygen saturation under both sauna and maximal exercise conditions (see Table 2). Similarly, the decreases in SaO_2_ during the sauna were smaller than those observed during exercise for all subjects, except for Subject 10, where levels remained unchanged, and Subject 4, where levels were increased during sauna.

#### 3.3.3. Muscle Oxygen Saturation Levels (SmO_2_)

In eleven subjects (Subjects 1, 2, 3, 5–11), SmO_2_ levels increased post-sauna compared to baseline, while in one subject they decreased (Subject 13) (see Table 2). Regarding maximal exercise, SmO_2_ levels decreased in ten subjects (Subjects 1–6, 9–12) compared to baseline, while they increased in two subjects (Subjects 7 and 8) (see Table 2).

#### 3.3.4. Hemoglobin Levels (Hb)

In six subjects (Subjects 1–3, 5, 11 and 12) hemoglobin levels increased post-sauna compared to baseline, and in the remaining six subjects, they decreased (Subjects 4, 6–10) (see Table 2). Regarding maximal exercise, hemoglobin levels increased in two subjects (Subjects 5 and 12) compared to baseline, but they decreased in ten subjects (Subjects 1–4, 6–11) (see Table 2).

#### 3.3.5. Glucose Values

In 11 subjects, glucose levels increased post-sauna compared to baseline values (see Table 2), while they remained unchanged in Subject 6. Regarding maximal exercise, all subjects showed an increase in glucose levels compared to baseline, being more pronounced than observed after sauna exposure (see Table 2).

#### 3.3.6. Total Cholesterol Levels

Total cholesterol levels increased post-sauna in six subjects (Subjects 1, 5, 6, 8, 11, and 12) from baseline levels, while they decreased in the remaining six subjects (2–4, 7, and 10) (see Table 2). Similarly, regarding maximal exercise, six subjects also had increased levels compared to baseline (Subjects 3, 5, 6, 8, 11, and 12), four had decreased levels (1, 4, 7, and 9) and two had levels that were unchanged (2 and 10) (see Table 2).

#### 3.3.7. Triglyceride Levels

In three subjects (1, 3, and 10), triglyceride levels increased post-sauna compared to baseline, while nine subjects had decreased levels (2, 4–9, 11, and 12) (see Table 2). Regarding maximal exercise, eight subjects showed an increase in triglyceride levels compared to baseline (Subjects 1–4, 6, 7, 9, and 10), while four subjects showed a decrease (5, 8, 11, and 12) (see Table 2).

#### 3.3.8. HDL Cholesterol Levels

All subjects (Subjects 1 to 12) showed an increase in HDL cholesterol levels both for sauna and maximal exercise conditions, except for Subject 8, whose levels decreased during sauna exposure (see Table 2). Similarly, the increases in HDL cholesterol during the sauna were smaller than those observed during exercise for all subjects (see Table 2).

#### 3.3.9. LDL Cholesterol Levels

All subjects (Subjects 1 to 12) showed a decrease in LDL cholesterol levels under maximal exercise conditions (see Table 2). During sauna exposure, LDL cholesterol levels increased in seven subjects (Subjects 5, 6, 8–12) and decreased in five subjects (Subjects 1–4 and 7) (see Table 2).

#### 3.3.10. Heart Rate Levels (HR)

All subjects (Subjects 1 to 12) showed an increase in heart rate under both sauna and maximal exercise conditions similarly; the HR increases during the sauna were lower than those observed during exercise for all subjects (see Table 2).

#### 3.3.11. Glucose and HDL Normalized Combined Parameters Levels

**Figure 2:**
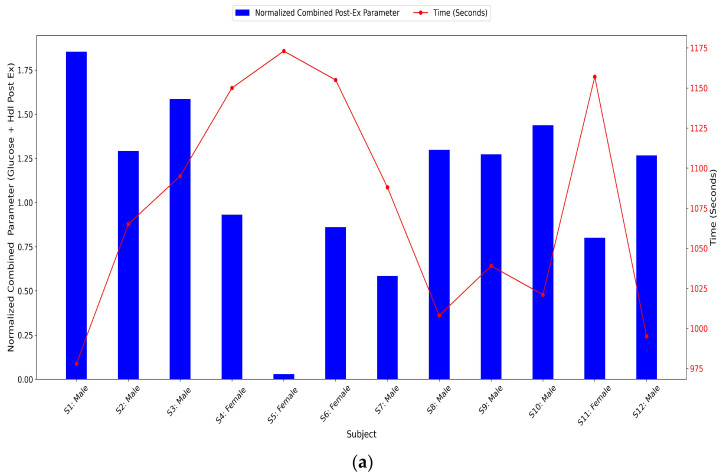

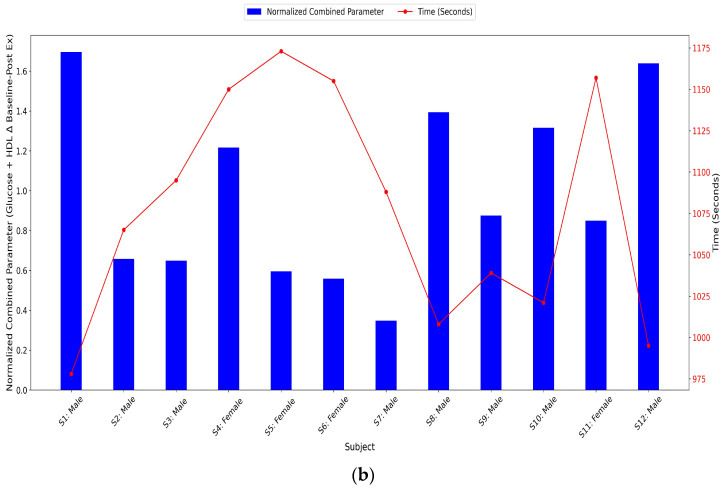
(**a**) Values of normalized combined parameters of glucose and HDL post-exercise (absolute post-exercise metabolic values) in all study participants (s: subject). (**b**) Values of normalized combined parameters of glucose and HDL between baseline values and post-exercise values in all study participants (s: subject).

**Figure 3:**
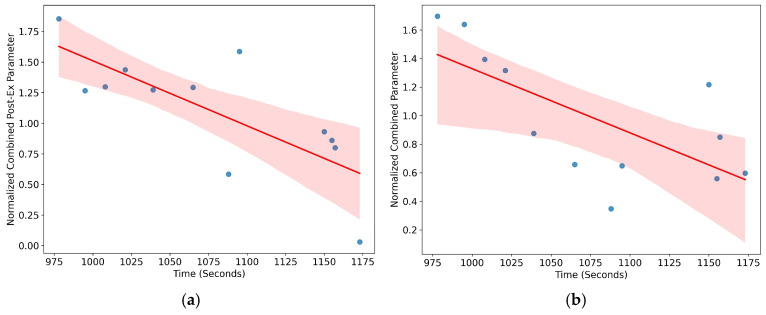
(**a**) Correlation between the sum of normalized post-exercise glucose and normalized post-exercise HDL (absolute post-exercise metabolic values) and running times in all subjects; (**b**) correlation between the normalized combined parameters of glucose and HDL (deltas between baseline and post-exercise values) and running times in all subjects.

In the post-exercise condition (absolute metabolic values), moderate to strong negative correlations between glucose and HDL were observed, reaching statistical significance in the general group (see Figure 2a and Figure 3a) (r = −0.76; *p* = 0.004), in delta values (see Figure 2b and Figure 3b) (r = −0.69; *p* = 0.012), and especially in male participants (delta male: r = −0.96; *p* < 0.001). In female participants, post-exercise correlation was also high (r = −0.95) and reached significance (*p* = 0.046), although the delta values were not statistically significant (*p* = 0.291).

In contrast, in the post-sauna condition, no statistically significant correlations between glucose and HDL were found, either in the general group or in the sex-based subgroups, despite some moderate correlations (e.g., delta female: r = −0.57).

## 4. Discussion

In the present study, we compared specific physiological responses (metabolism, oxygenation, and heart rate) in a group of elite endurance athletes under two conditions: passive heat sauna and maximal distance running (see Figure 1). The results indicated that acute hyperthermic stimuli (sauna exposure) moderately increases physiological demands in comparison with their basal state and maximal exercise activity. It should be noted that certain physiological variables such as glycemia, HDL cholesterol, and systemic oxygen kinetics changed in a similar manner both for sauna and exercise (see Figure 1 and Table 1 and Table 2). The main effect promoted by the sauna protocol (20 min at 80–90 °C) was associated with a rapid elevation of the core body temperature (mean: 39.4 °C). The maximal peak of the thermic response achieved during the sauna was similarly found during maximal exercise, which is indicative of a peak temperature threshold. The rapid increase in temperature-related effects of acute sauna exposure altered both metabolic and oxygenation responses (see Table 2) and triggered rapid predictive heat loss responses such as vasodilation and sweating [27]. The average increase of 2.8 °C in tympanic temperature during sauna exposure resulted in profuse sweating with average fluid losses of 400–600 g, which was consistent with similar studies [28]. During maximal exercise, deltas combining substrate mobilization (glucose and HDL cholesterol) indicated that the athletes with better endurance performances (shorter running time) were those who exhibited greater combined increases in glucose and HDL (r = −0.69; *p* = 0.012), especially in the male delta group (r = −0.96; *p* = 0.0001). This overall trend becomes even more pronounced when considering absolute post-exercise values (r = −0.76; *p* = 0.004) (see Figure 2a,b and Figure 3). However, this response was not observed during sauna exposure, confirming the moderate effect on metabolic response from passive conditions (r = −0.49; *p* = 0.22).

Previous studies have demonstrated that repeated exposure to passive hyperthermia can contribute to improve aerobic efficiency and effort tolerance during heat events in athletes [29]. Here, we confirm that only one instance of passive sauna exposure induced moderate changes at metabolic and oxygen levels but also resulted in lower stress compared with exercise (see Figure 1 and Table 1 and Table 2). Sauna exposure caused significant glycemia elevation in 11 of 12 athletes (ranging from 4% to 37%) (see Table 2). For lipid mobilization, total cholesterol levels showed significant variations, with 6 out of 12 athletes experiencing increases of up to 57% and 6 exhibiting decreases ranging from 7% to 16% (see Table 2). The levels of TG significantly decreased in most athletes (9 out of 12) (see Table 2), while LDL cholesterol levels showed varied individual responses (see Table 2). The elevation of glucose observed in the present study could be linked to an increase in stress hormones during acute heat exposure, such as plasma noradrenaline and cortisol [30]. In the absence of muscle contraction as occurred during sauna exposure, the specific mechanism underlying this effect remains unclear, although it is possibly driven by the activation of hepatic gluconeogenesis, which may elevate fasting plasma glucose levels. The increased peripheral vasodilation and blood flow to muscles could potentially activate metabolic responses in a dose-dependent manner. This hypothesis was confirmed here with the observed elevation of muscle oxygen saturation during sauna exposure (see Figure 1 and Table 1 and Table 2).

Regarding the lipid profile response to sauna exposure, previous studies reported increases in total cholesterol and LDL levels compared to controls [31,32]. Conversely, some studies demonstrated no effects of sauna on TG levels [33], while others reported an elevation following a single sauna session [32]. In contrast, our results found that TG levels decreased significantly (*p* = 0.03) in the group of athletes after the sauna but showed a slight, non-significant increase after exercise. It is plausible that a single session of heat exposure is insufficient to significantly alter chronic metabolic responses and longer exposure periods may be required to promote adaptive mechanisms, as was observed in previous studies. In this context, Horowitz and Klein [34] demonstrated that TG oxidation progressively increases during exercise, presumably from intramuscular sources. Similarly, our research group confirmed that TG levels progressively rise during an ultra-endurance race of 170 km [18], as observed in some athletes in the present study following maximal exercise ((a) i.e., a 9.98% average increase (see Table 1)). However, the sauna stimulus promoted a group reduction of 12.7%. The average lipid elevations during exercise were 10% for TG and 3.4% for cholesterol, whereas sauna exposure increased cholesterol by 1.2% but reduced TG levels by 12.7% (see Table 1). The elevation of catecholamines during exercise may explain the increase in lipids and their mobilization, which did not occur during passive sauna stress [34].

The effects of sauna and exercise on HDL and LDL cholesterol were similar for HDL trends but opposite for LDL (see Table 1 and Table 2). Sauna increased the average of HDL levels by 23.6% and LDL levels by 26.5%, while exercise significantly increased HDL levels by 133% but reduced LDL levels by 47.2%. Moreover, it was found that exercise significantly increased HDL levels (*p* = 0.0001) (see Table 1), while sauna showed a smaller but still significant increase (*p* = 0.023). Higher levels of HDL mobilized during exercise have been associated with a better endurance capacity [35]. Therefore, the present results confirmed that high-intensity exercise can be an optimal stimulus for increasing HDL levels and reducing LDL levels in elite athletes. From a systemic health perspective, the metabolic changes observed here confer multiple benefits, including anti-inflammatory, antioxidant, and antithrombotic effects [36]. Previous studies have postulated that increases in HDL and reductions in LDL after exercise are attributed to the regulatory action of lipoprotein lipase [35]. In this context, the chronic effects that endurance exercise promotes in enhanced lipoprotein profile expression could be increased through the addition of sauna by contributing lipid mobilization. The differences in LDL levels during sauna and exercise (increase after sauna but decrease after exercise) could be related to minimal muscular activity and a lack of energy uptake in the passive sauna exposure.

Sauna exposure moderately increased heart rate levels (see Table 1) like low aerobic exercise (e.g., fast walking). Previous studies have reported that an increase of 1 °C in internal temperature due to passive hyperthermia is equivalent to an increase of 7 to 8 beats per min [37,38]. Our results confirmed that during sauna and exercise, athletes increased their temperature by 2.8 °C and by 21 to 46 beats per min per 1 °C of temperature increase, respectively. Additionally, exercise-promoted peak skin temperatures like those reached during sauna exposure (39.4) were experienced in unheated conditions (12–22 °C) with light clothing, allowing for evaporative cooling. Consequently, the temperature thresholds that increased slowly during exercise were related to the energy production of running, unlike in the sauna where the increase was related to the heat exposure. Therefore, the sauna promoted the faster activation of thermoregulatory mechanisms and was not reliant on energy production related to the predictive responses of thermoneutrality and heat dissipation. Despite this, 5 out of 12 subjects reached higher temperature limits during exercise compared to in the sauna, while the remaining subjects experienced sauna-induced temperature increases of 0.1 °C to 1.7 °C over exercise.

Arterial and muscular oxygenation decreased significantly during maximal exercise (*p* < 0.001 and *p* < 0.04) (see Table 1), while the sauna increased SmO_2_ values and decreased SaO_2_ (*p* < 0.05). The reduction in SmO_2_ during exercise may be connected to Hb decreases, as hemoglobin supplies oxygen to active muscles. The Hb levels decreased significantly by 8.2% (*p* < 0.025) compared to baseline values, likely due to active muscle demands for oxygen uptake and their extraction from the capillaries [39]. These Hb decreases, ranging from 5% to 21%, occurred in 10 out of 12 subjects, while Subject 5 showed an increase of 18%. In contrast to exercise, sauna exposure increased SmO_2_ while Hb remained stable, possibly due to heat-induced vasodilation in the peripheral tissues and related to the lower oxygen demand of muscles in the absence of contraction. In this regard, Schenaarts et al. [30] speculated that increased muscle perfusion during heat stress primarily facilitates heat dissipation to the skin rather than enhancing peripher1al glucose uptake in skeletal muscle. On the other hand, another hypothesis could be that heat induces respiratory alkalosis and decreases hemoglobin binding capacity to venous blood oxygen, thereby facilitating oxygen transport into body tissues [40]. Here, we found that hemoglobin levels after sauna exposure were comparable to baseline values (ns), although some subjects exhibited acute significant changes (see Table 2). For example, the Hb levels of Subjects 4 and 9 decreased by 10% and 14% after sauna exposure, respectively, while Subject 5 had Hb levels increased by 42%.

### 4.1. Potential of Passive Heat Stimuli

Passive hyperthermic stimuli (HS) could have the potential to promote moderate systemic responses related to metabolic flexibility, cardiovascular, and oxygenation responses with moderate effects improving adaptive processes and physical performance. Although the literature suggests that dry saunas may mimic the physiological and protective responses induced during moderate to vigorous aerobic exercise [41], our results indicated that these responses do not manifest similarly in elite endurance athletes. In this group, the responses to passive heat exposure are more comparable to those induced by low-intensity aerobic exercise and dose-dependent manner related to metabolic activation. It is likely that the benefits attributed to sauna as a hormetic stressor—promoting the increased expression of heat shock proteins [42], transcriptional regulators [43], and pro- and anti-inflammatory factors [44]—observed in previous studies, would be influenced by the characteristics of the studied populations, which predominantly include healthy individuals with moderate physical fitness [45] or individuals with pathologies or physical limitations [44,46,47]. This underscores the need to tailor heat exposure strategies to the physiological profile and training level of each population and redefine the environmental stimuli as systemic biological effects to maintain the physiological inertia of exercise training and routines. In this regard, the biological potentiality of passive heat stimuli could be associated with maintaining physiological stimuli like moderate exercise in athletes during phases of recovery and/or injury. It is worth noting that metabolic flexibility is particularly relevant to performance in endurance athletes, as we observed that the athletes with better performance were those who showed significantly greater combined increases in glucose and HDL. This suggests that an organism’s capacity to mobilize and efficiently utilize different energy substrates in response to a thermal or physical stimulus could be a key marker of the level of physiometabolic adaptation in this group of athletes. Moreover, the benefits of passive heat exposure may be valuable in other contexts and for instance, it could benefit individuals with good physiological status or athletes unable to perform physical training due to musculoskeletal injuries, as sauna exposure provides physiological stimuli similar to those of exercise [42]. Furthermore, a promising strategy could involve performing low-intensity exercise under controlled hyperthermic conditions, enabling systemic training—particularly at cardiovascular, renal, and endocrine-immune levels—while preserving other systems still in the process of recovery. Moreover, the observed physiological changes could be employed to reinforce the potentiality of exercise stimuli because these may be linked to genetic responses that prolong protein and inflammatory pathways similar to endurance exercise [48]. These observations reinforce the argument for individual variability in responses to hyperthermic stimuli and the necessity of personalizing interventions. This study opens a pathway for future research and scientific debate on the relationship between passive heat responses and individual physiological trends observed during maximal exercise. Such investigations could contribute to the design of more individualized training protocols under hyperthermic conditions, with tailored dosing and progression to maximize efficacy and specific benefits according to a subject’s characteristics.

### 4.2. Limitations

This study was conducted with a small sample of elite athletes and focused on analyzing an acute hyperthermia stimulus, which limits the possibility of assessing long-term adaptive capacity, unlike the responses observed in chronic exercise among high-performance athletes. The main problem encountered in this study was the difficulty of obtaining biological samples and analyzing the physiology of professional athletes, which led to an increase in the number of participants. Additionally, average group metabolic responses cannot be directly extrapolated to individual changes due to inherent phenotypic variability among participants.

The results provide valuable insights into the acute effects of sauna exposure on physiological responses but do not fully determine how passive heat stimuli may influence adaptive mechanisms or promote faster or slower responses during endurance exercise in heated conditions. To address these limitations, it would be useful to measure individual recovery curves for variables such as temperature, oxygenation, and metabolic parameters, which could help better identify responders to heat and exercise.

Moreover, the physical activity used as a test in this study was performed under normothermic conditions, making it difficult to generalize the findings to heat stress scenarios. Evaluating physiological responses during exercise in extreme heat environments would provide more specific and relevant data, enabling a deeper understanding of the metabolic and systemic adaptations that occur under thermally demanding conditions.

## 5. Conclusions

The present study explored the hypothesis that for elite endurance athletes, passive heat exposure could provide insights into individual physiological responses like those occurring during light to moderate exercise but not during maximal activity. Much of the extant research focuses on non-athletic populations or individuals with physical limitations, which restricts its applicability to elite endurance athletes. We observed that sauna exposure at 80 °C increased moderately average levels of heart rate, oxygenation kinetics, and metabolic responses with remarkably individuality. Therefore, it would be necessary to propose a comparative individual analysis of physiological responses to heat stimulus and maximal exercise for specific metabolic, oxygenation, and thermic kinetics. Maximal endurance exercise reflects an individual phenotype under peak systemic demands, which are not achieved during passive sauna exposure for some characteristics. The results suggest that passive heat exposure generates responses like biological changes occurred during low-intensity exercise in endurance athletes. Therefore, the present results are consistent with the idea that passive heat stimuli promote a reduced level of physiological disruption in highly trained individuals and could promote an alternative strategy of combining low-intensity exercise in controlled hyperthermic conditions, which is promising as it could promote specific adaptations without compromising the primary training load. Notably, certain physiological variables, such as SaO_2_, peak temperature, and the kinetics of glucose and HDL cholesterol increases, may indicate individual responses to different environmental and exercise stimuli. However, other variables, including TG, Hb, and LDL cholesterol, followed different trends after sauna exposure compared to exercise. Based on the observed correlations between glucose and HDL levels during sauna and maximal exercise, measuring these parameters could be useful in defining the metabolic endurance phenotype. Although it was not the main goal of the present study, we found that greater substrate mobilization during maximal endurance exercise was significantly correlated with better performance. In this regard, including repeated passive heat stimuli could contribute to improving metabolic flexibility in trained endurance athletes. These findings could lay the groundwork for longitudinal studies that explore how passive heat exposure, combined with controlled exercise, may influence aspects such as thermal stress resilience, recovery capacity, and metabolic efficiency. The present results demonstrated that greater substrate mobilization involving glucose and HDL cholesterol could improve maximal endurance performance in athletes and is a direct description of metabolic flexibility.

## Figures and Tables

**Table 1:** Statistical data for baseline (resting conditions), sauna (maximal values), post-sauna (immediately after leaving the sauna chamber), and post-exercise (maximal physiological responses measured during exercise).

Variable	Baseline	Sauna	Post-Sauna	Post-Exercise
Age	32.5 ± 6.8			
Height (cm)	168.7 ± 8.2			
Weight (kg)	59.8 ± 8.3		59.3 ± 8.4 **	
BMI	21.01		20.84	
Temperature (°C)	36.6 ± 0.3	39.4 ± 0.4 *		39.4 ± 0.5 *
SaO_2_ (%)	98.8 ± 0.7	91.3 ± 3.1 **		88.9 ± 3.5 **
SmO_2_ (%)	53.8 ± 13.8	58.9 ± 16.8 **		43.4 ± 9.1 NS
Hb (g/dL)	14.6 ± 1.4		14.6 ± 1.2 NS	13.4 ± 0.9 *
Glucose (mg/dL)	84.7 ± 4.6		99.2 ± 6.8 **	123.7 ± 11.8 **
TC (mg/dL)	190.2 ± 45.5		192.4 ± 40.1 NS	196.7 ± 49.5 NS
TG (mg/dL)	102.2 ± 20.1		89.2 ± 19.3 *	112.4 ± 27.8 NS
HDL Chol (mg/dL)	73.8 ± 20		91.2 ± 17.1*	172.2 ± 17 *
LDL Chol (mg/dL)	68.8		87 ± 16 NS	36.3 * ± 8.9 **
HR (beats/min)	50.7 ± 8.9	112.2 ± 14.1 **		176.2 ± 6.6**
BL (mmol/L)	1.1 ± 0.5		1.75 ± 0.4	11.5 ± 1.9 **

Significance levels are indicated either as significant (* *p* ≤ 0.05 and ** *p* ≤ 0.001) or non-significant (NS, *p* > 0.05) for each condition. Abbreviations used: BMI—body mass index; SaO_2_—% arterial oxygen saturation; SmO_2_—% muscular oxygen saturation; Hb—hemoglobin; TC—total cholesterol; TG—triglycerides; HDL and LDL—high- and low-density lipoproteins of cholesterol; HR—heart rate; BL—blood lactate.

**Table 2:** Statistical data for baseline (resting conditions), post-sauna, and post-exercise values.

Variable		T (°C)	SaO_2_ (%)	SmO_2_ (%)	Hb (g/dL)	GL (mg/dL)	TC (mg/dL)	TG (mg/dL)	HDL (mg/dL)	LDL (mg/dL)	HR (bpm)	BL (mmol/L)	WL (g)	Time (s)
S1	B	36.4	98	63.1	15.1	77	250	110	77	56	52	0.7	300	978
	S	39.7	90	66.8	15.8	96	268	119	99	50	130	1.9		
	E	39.1	89	44	14.4	136	233	131	184	46	181	14.1		
S2	B	36.8	99	60.2	15	80	260	99	101	88	44	1.1	600	1065
	S	39.8	88	65.7	15.4	106	233	80	109	86	112	2.1		
	E	39.7	86	52	13.6	126	258	118	176	52	177	12.7		
S3	B	36.6	100	66.8	14.2	92	240	80	99	106	40	1.6	590	1095
	S	38.9	91	72.3	14.4	109	218	114	106	101	121	1.9		
	E	39.8	90	44	13.1	136	242	107	176	36	173	13.3		
S4	B	36.2	99	62.1	12.9	81	220	77	60	68	46	1.1	700	1150
	S	39.4	88	70.2	11.7	111	203	60	102	58	102	1.7		
	E	38.9	89	52	11.2	116	207	99	174	41	170	10.3		
S5	B	36.4	99	62.5	11.3	85	113	139	54	79	63	1.2	500	1173
	S	39.6	89	76	16.1	98	127	115	59	143	100	1.1		
	E	39.3	80	52	13.4	108	198	88	154	39	175	11.6		
S6	B	37.3	100	65.2	16.4	88	175	84	80	78	51	1.3	400	1155
	S	38.6	97	71	16	88	194	78	92	86	97	1		
	E	39.7	95	43	13	117	231	112	171	36	195	12.5		
S7	B	36.5	98	35.7	15.7	90	166	99	77	64	51	1.5	500	1088
	S	39.7	92	37.8	14.6	92	141	75	78	62	116	1.7		
	E	39.3	90	39	14.5	111	104	183	168	42	176	10.8		
S8	B	37.1	99	35.6	15.1	83	156	116	66	54	45	1.2	400	1008
	S	39.8	97	36.9	13.7	100	200	86	60	123	118	2.4		
	E	39.6	91	52	13.8	133	199	75	170	20	173	9.7		
S9	B	36.9	99	32.2	15.8	85	179	111	86	33	70	1.2	200	1039
	S	39.4	94	36.1	13.5	98	150	70	90	60	82	1.7		
	E	39.8	90	28	14.1	131	100	135	171	23	176	7.7		
S10	B	36.6	98	38.2	15	90	211	77	62	82	52	1.5	400	1021
	S	39.9	89	64.2	14.7	101	189	90	100	89	122	1.6		
	E	38.2	89	31	13.8	123	211	99	183	35	172	9.9		
S11	B	36.4	98	64.2	14.7	81	147	108	70	60	40	1.1	300	1157
	S	39.1	91	72	15.2	99	212	103	107	105	119	1.9		
	E	39.3	90	52	12	107	178	102	178	30	175	13.4		
S12	B	36.2	99	60.1	13.7	85	166	127	54	58	55	1.2	300	995
	S	38.9	90	37.8	14.3	93	174	80	93	81	127	2.1		
	E	39.6	88	32.1	14.3	141	199	100	162	36	171	12.2		

Abbreviations used: B—baseline values; S—post sauna values; E—post exercise values; T—tympanic temperature; SaO_2_—% arterial oxygen saturation; SmO_2_—% muscular oxygen saturation; Hb—hemoglobin; GL—glucose; TC—total cholesterol; TG—triglycerides; HDL and LDL—high- and low-density lipoproteins of cholesterol; HR—heart rate; BL—blood lactate; WL—weight loss; Time—trail running test completion time.

## Data Availability

Data is contained within the article.
